# A high seroprevalence of antibodies to pertussis toxin among Japanese adults: Qualitative and quantitative analyses

**DOI:** 10.1371/journal.pone.0181181

**Published:** 2017-07-10

**Authors:** Takumi Moriuchi, Nao Otsuka, Yukihiro Hiramatsu, Keigo Shibayama, Kazunari Kamachi

**Affiliations:** 1 Department of Bacteriology II, National Institute of Infectious Diseases, Tokyo, Japan; 2 Department of Pediatrics, St Marianna University School of Medicine, Kawasaki, Japan; Universidad Nacional de la Plata, ARGENTINA

## Abstract

In 2013, national serosurveillance detected a high seroprevalence of antibodies to pertussis toxin (PT) from *Bordetella pertussis* among Japanese adults. Thus, we aimed to determine the cause(s) of this high seroprevalence, and analyzed the titers of antibodies to PT and filamentous hemagglutinin (FHA) among adults (35–44 years old), young children (4–7 years old), and older children (10–14 years old). Our quantitative analyses revealed that adults had higher seroprevalences of anti-PT IgG and PT-neutralizing antibodies, and similar titers of anti-FHA IgG, compared to the young and older children. Positive correlations were observed between the titers of PT-neutralizing antibodies and anti-PT IgG in all age groups (r_s_ values of 0.326–0.522), although the correlation tended to decrease with age. The ratio of PT-neutralizing antibodies to anti-PT IgG was significantly different when we compared the serum and purified IgG fractions among adults (*p* = 0.016), although this result was not observed among young and older children. Thus, it appears that some adults had non-IgG immunoglobulins to PT. Our analyses also revealed that adults had high-avidity anti-PT IgG (avidity index: 63.5%, similar results were observed among the children); however, the adults had lower-avidity anti-FHA IgG (37.9%, *p* < 0.05). It is possible that low-avidity anti-FHA IgG is related to infection with other respiratory pathogens (e.g., *Bordetella parapertussis*, *Haemophilus influenzae*, or *Mycoplasma pneumoniae*), which produces antibodies to FHA-like proteins. Our observations suggest that these adults had been infected with *B*. *pertussis* and other pathogen(s) during their adulthood.

## Introduction

Pertussis (whooping cough) is a major acute respiratory infection that is caused by the bacterial pathogen *Bordetella pertussis*, and is associated with severe respiratory illness among children and a persistent cough among adolescents and adults. Adolescents and adults are the primary reservoir for this pathogen, and play a crucial role in transmission to infants and unvaccinated children [1, 2]. Immunization is the most effective method for preventing and controlling pertussis, although the incidence of pertussis has increased in several industrialized countries, despite their high vaccination coverage rates [3, 4]. In Japan, pertussis vaccination was introduced in 1950 using whole-cell pertussis vaccines according to a 4-dose schedule (3 primary doses and a single booster dose) starting at 3 months of age. In 1981, acellular pertussis vaccines (ACVs) were introduced, using a 4-dose schedule beginning at 2 years of age. Since 1994, ACVs have been used according to a schedule of 3 primary doses (at the ages of 3, 4, and 6 months) and a single booster dose (at the age of 18–23 months). However, since the early 2000s, the incidence of pertussis among adolescents and adults has significantly increased in Japan [5], and previous studies have demonstrated that waning immunity after receiving ACVs is a major factor that has contributed to the recent resurgence of pertussis [6, 7].

*B*. *pertussis* produces several virulence factors (e.g., toxins and adhesins), and pertussis toxin (PT) and filamentous hemagglutinin (FHA) are included as major antigens in ACVs. Therefore, antibodies to PT and FHA are generated by both *B*. *pertussis* infection and immunization. Previous studies have evaluated the titers of anti-FHA and anti-PT antibodies (especially anti-PT IgG) among various age groups in many countries [8–12], and revealed that vaccine-induced anti-PT IgG titers rapidly waned over time. Furthermore, *B*. *pertussis* infection persists among adolescents and adults in countries with high vaccination coverage rates [10–14]. Interestingly, the seroprevalence of anti-FHA IgG is higher than that of anti-PT IgG in various age groups [13, 14].

To monitor vaccine-induced herd immunity in Japan, national pertussis serosurveillance has been implemented every 5 years by the National Epidemiological Surveillance of Vaccine-Preventable Disease (NESVPD, http://www.nih.go.jp/niid/ja/y-graphs/1600-yosoku-index-e.html). A 1994 surveillance study revealed a bimodal distribution of anti-PT IgG titers according to age (peaks at age of 3–5 years and 10–19 years), despite nearly constant distributions of anti-FHA IgG titers between the ages of 3 years and 19 years [15]. The most recent serosurveillance study (2013) revealed a significant increase in the seroprevalence of elevated anti-PT IgG titers [≥10 ELISA units (EU)/mL] among adults who were >30 years old, compared to serosurveillance data from 2008 (an increase from 39% to 77%). In contrast, there was only a small change in the seroprevalence of elevated anti-FHA IgG titers (≥10 EU/mL) in that population (from 70% in 2008 to 72% in 2013). Thus, although pertussis serosurveillance remains essential to monitor the current immunization program, it remains unclear what factor(s) caused the increase in the seroprevalence of elevated anti-PT IgG titers among Japanese adults.

Pertussis antibodies to PT and FHA are commonly assessed by quantifying serum titers of IgG antibodies. In addition to the quantitative assay, a Chinese hamster ovary (CHO) cell clustering assay can be used to measure PT-neutralizing antibodies, which avidly bind to and neutralize PT [16, 17]. In addition to detecting anti-PT IgG, the CHO cell clustering assay might also detect non-IgG immunoglobulins (e.g., anti-PT IgM and anti-PT IgA); this can be used as a qualitative measure of PT antibodies. The avidity of anti-PT IgG and anti-FHA IgG for their antigen can also be measured using enzyme-linked immunosorbent assays (ELISA) with dissociating agents [18–20]. This avidity ELISA measures the strength of antibody–antigen binding, thereby providing a measure of the quality of the antibodies. In the present study, we performed both a quantitative (ELISA) and qualitative (CHO cell clustering assay and avidity ELISA) analysis of pertussis antibodies obtained from the serum samples of children and adults, in order to identify factors that might have caused the increased seroprevalence of anti-PT IgG among adults in the 2013 NESVPD.

## Materials and methods

### Serum samples

A total of 252 serum samples (40 μL each) were obtained from the National Serum Reference Bank of the National Institute of Infectious Diseases (Tokyo, Japan). The samples had been collected from 252 healthy subjects in three age groups (4–7 years, 10–14 years, and 35–44 years) from 6 prefectures during 2013–2014 (48 samples in 2013 and 36 samples in 2014 for each age group). The samples were heated at 56°C for 30 min and stored at 4°C until use. The National Serum Reference Bank of the National Institute of Infectious Diseases prohibits the handling of personal information (except for age, sex, residential area, and data of serum collection); hence, the subjects’ medical and vaccination histories, considered personal information, were not available. The present study’s design was reviewed and approved by the Human Ethics Committee of the National Institute of Infectious Diseases.

### Purification of IgG fractions from the serum samples

A total of 108 serum samples from 2014 were subjected to IgG purification. The IgG fractions were purified from 20 μL of each serum sample using protein G spin columns (MonoSpin Pro G; GL Sciences Inc., Tokyo, Japan), according to the manufacturer’s instructions. The purified IgG fractions (approximately 550 μL) were dialyzed against phosphate-buffered saline (PBS) using Amicon Ultra-0.5 centrifugal filters (100K; Merck Millipore, Germany), concentrated to a final volume of approximately 20 μL, and stored at –20°C until use.

### In-house ELISA for anti-PT IgG and anti-FHA IgG

Titers of anti-PT and anti-FHA IgG were measured using an in-house ELISA that was adapted to test heat-treated serum samples [21]. We coated 96-well ELISA plates (Nunc MaxiSorp) with PT or FHA (100 ng/50 μL/well, Seikagaku Corp., Japan), and washed the plates using PBS with 0.1% Tween 20 (PBST). After blocking with 1% skim milk (Difco Laboratories, USA) in PBST, 100 μL of the serum or IgG fraction (diluted 1:200 using 0.1% skim milk in PBST) was added and incubated for 2 h at 36°C. After washing with PBST, the bound antibodies were incubated with alkaline phosphatase-labeled rabbit anti-human immunoglobulin G (Dako, Denmark), and *p*-nitrophenyl phosphate was used to develop the reaction. The absorbance at 405 nm was measured using the Multiskan FC Microplate Photometer (Thermo Scientific, Waltham, MA), and the reference wavelength was 650 nm. Japanese human reference serum (JNIH-10) was used as the working standard (250 EU/mL for anti-PT IgG and 400 EU/mL for anti-FHA IgG) [22], and the antibody titers were calculated in international units (IU)/mL using the following unit conversions: anti-PT IgG, IU/mL = 1.19 × EU/mL [23]; anti-FHA IgG, IU/mL = 1.01 × EU/mL (data not shown).

### CHO cell clustering assay for PT-neutralizing antibodies

The CHO cell clustering assay was used to evaluate PT-neutralizing antibodies [24]. The serum or IgG fractions were diluted 1:10 using PBS, and then the samples were serially diluted (1:3 increments) in 96-well plates (range: 1:10 to 1:21,870). The dilution series (30 μL/well) was mixed with 30 μL of PT solution (2 ng/mL in PBS with 0.2% gelatine) and incubated for 30 min at 36°C. After the incubation, 50 μL of each dilution was added to CHO cells (1 × 10^4^ cells/50 μL/well) that had been cultured for 4 h with Ham’s F12 medium containing 10% fetal calf serum in 96-well tissue culture plates. The plates were incubated for 22–24 h at 37°C in a 5% CO_2_ incubator, and clustering of the cells was evaluated using a phase-contrast microscope. Neutralization titers were expressed as the reciprocal of the highest sample dilution that provided nearly 100% neutralization of PT’s clustering effect.

The CHO cell clustering assay for PT-neutralizing antibodies was highly reproducible when three independent measures were employed for the reference sera (S1 Fig).

### Avidity ELISA for anti-PT IgG and anti-FHA IgG

The avidity values for anti-PT IgG and anti-FHA IgG were measured using IgG fractions and NH_4_SCN as the dissociating agent [18, 19]. The avidity assay was performed in the same manner as our in-house ELISA, and an NH_4_SCN incubation step was added after the IgG incubation. The IgG fraction (1:200 dilution) was applied in duplicate wells of an ELISA plate. After incubation with the IgG fraction, the wells were washed three times using PBST, and 100 μL of 1.5 M NH_4_SCN or PBS were added to the wells. The wells were incubated for 20 min at 37°C, washed five times using PBST, and processed in the same manner as for the in-house ELISA. The avidity index (AI) was calculated as: ([sample OD_405_ –blank OD_405_ in the presence of NH_4_SCN] / [sample OD_405_ –blank OD_405_ in the absence of NH_4_SCN]) × 100%.

The avidity ELISA was highly reproducible when three independent measures were employed for the reference sera (S2 Fig). The maximum coefficients of variation were 8.1% and 13.3% for anti-PT IgG and anti-FHA IgG, respectively.

### Statistical analyses

Fisher’s exact test was used for evaluating categorical variables, the seroprevalence rates, and the antibody ratios (PT-neutralizing antibodies/anti-PT IgG). The nonparametric Steel-Dwass test for multiple comparisons was used to evaluate differences in antibody avidity according to age. *P*-values of <0.05 were considered statistically significant. Spearman’s rank correlation coefficients were used to evaluate the correlations between anti-PT IgG and PT-neutralizing antibody titers.

## Results

### Seroprevalences of pertussis antibodies according to age

Table 1 summarizes the geometric mean concentrations of anti-PT IgG and anti-FHA IgG among the different age groups. The mean anti-PT IgG titers were highest among the adults (35–44 years old, 58.4 IU/mL) and lowest among the young children (4–7 years old, 43.6 IU/mL). The mean anti-FHA IgG titers were highest among the older children (10–14 years old, 29.9 IU/mL). In 2016, the guidelines committee of the Japanese Society for Pediatric Infectious Diseases proposed a cut-off value of 100 EU/mL (corresponding to 119 IU/mL) PT IgG to classify possible cases of *B*. *pertussis* infection.

**Table 1:** Geometric mean serum concentrations of anti-PT and anti-FHA IgG among young children, older children, and adults during 2013–2014 in Japan.

Age group	Mean age (years)	No. of serum samples^a^	IU/mL (95% CI)	
			anti-PT IgG	anti-FHA IgG
Young children (4–7 years old)	5.6	84	43.6 (29.2–64.9)	23.0 (6.4–82.8)
Older children (10–14 years old)	11.7	84	54.1 (34.9–83.6)^b^	29.9 (10.8–82.3)
Adults (35–44 years old)	38.9	84	58.4 (41.0–83.3)	22.3 (10.1–49.5)

^a^ Forty-eight and 36 serum samples were collected in 2013 and 2014, respectively.

^b^ Data were obtained from 82 serum samples (48 in 2013 and 34 in 2014). Two serum samples were not analyzed because of an insufficient sample volume.

**Fig 1:**
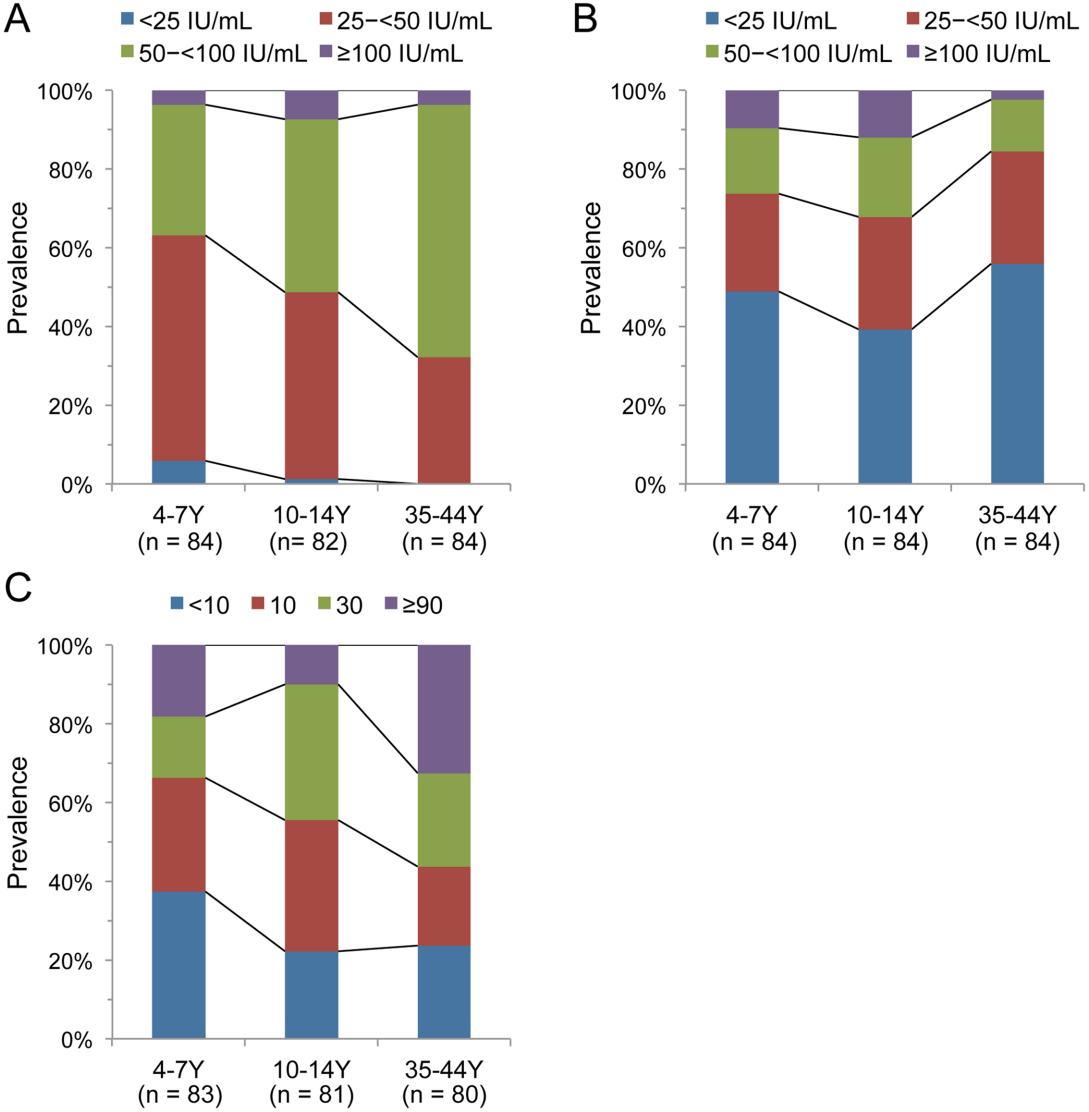
Seroprevalence of pertussis antibodies among young children, older children, and adults during 2013–2014 in Japan. (A) anti-PT IgG, (B) anti-FHA IgG, (C) PT-neutralizing antibodies (the highest reciprocal serum dilution that was needed to neutralize the ability of PT to induce Chinese hamster ovary cell clustering). The age groups were 4–7 years, 10–14 years, and 35–44 years. Numbers in parentheses indicate the number of serum samples that were analyzed. Y, years.

In the present study, some serum samples were not analyzed because of the small sample volume and/or cytotoxicity in the CHO cells. When the available serum samples were classified according to titer (<25 IU/mL, 25–<50 IU/mL, 50–<100 IU/mL, and ≥100 IU/mL), significant age-related differences were observed in the prevalences of anti-PT IgG (*p* < 0.01) (Fig 1A). The young and older children had the highest proportions of anti-PT IgG at 25–<50 IU/mL (57.1% and 47.6%, respectively), whereas the adults had the highest proportion of anti-PT IgG at 50–<100 IU/mL (64.3%). The prevalence of anti-PT IgG at 50–<100 IU/mL increased with age. In contrast, the prevalences of anti-FHA IgG did not exhibit significant age-related differences (*p* > 0.05) (Fig 1B). The highest proportions of anti-FHA IgG were observed at <25 IU/mL for the young children, older children, and adults (48.8%, 39.3%, and 56.0%, respectively). Next, we evaluated PT-neutralizing antibodies using the CHO cell clustering assay. Fig 1C shows the prevalences of PT-neutralizing antibodies, which exhibited significantly age-related differences (*p* < 0.01). Young children had the highest proportion of PT-neutralizing antibodies at titers of <10 (37.3%), whereas older children and adults had the highest proportions of titers at 30 (34.6%) and ≥90 (32.5%), respectively. The prevalence of PT-neutralizing antibody titers of ≥30 increased with age (young children, 33.7%; older children, 44.4%; adults, 56.3%), which was similar to the findings for the prevalence of elevated anti-PT IgG titers.

### Correlations between anti-PT IgG and PT-neutralizing antibody titers

**Fig 2:**
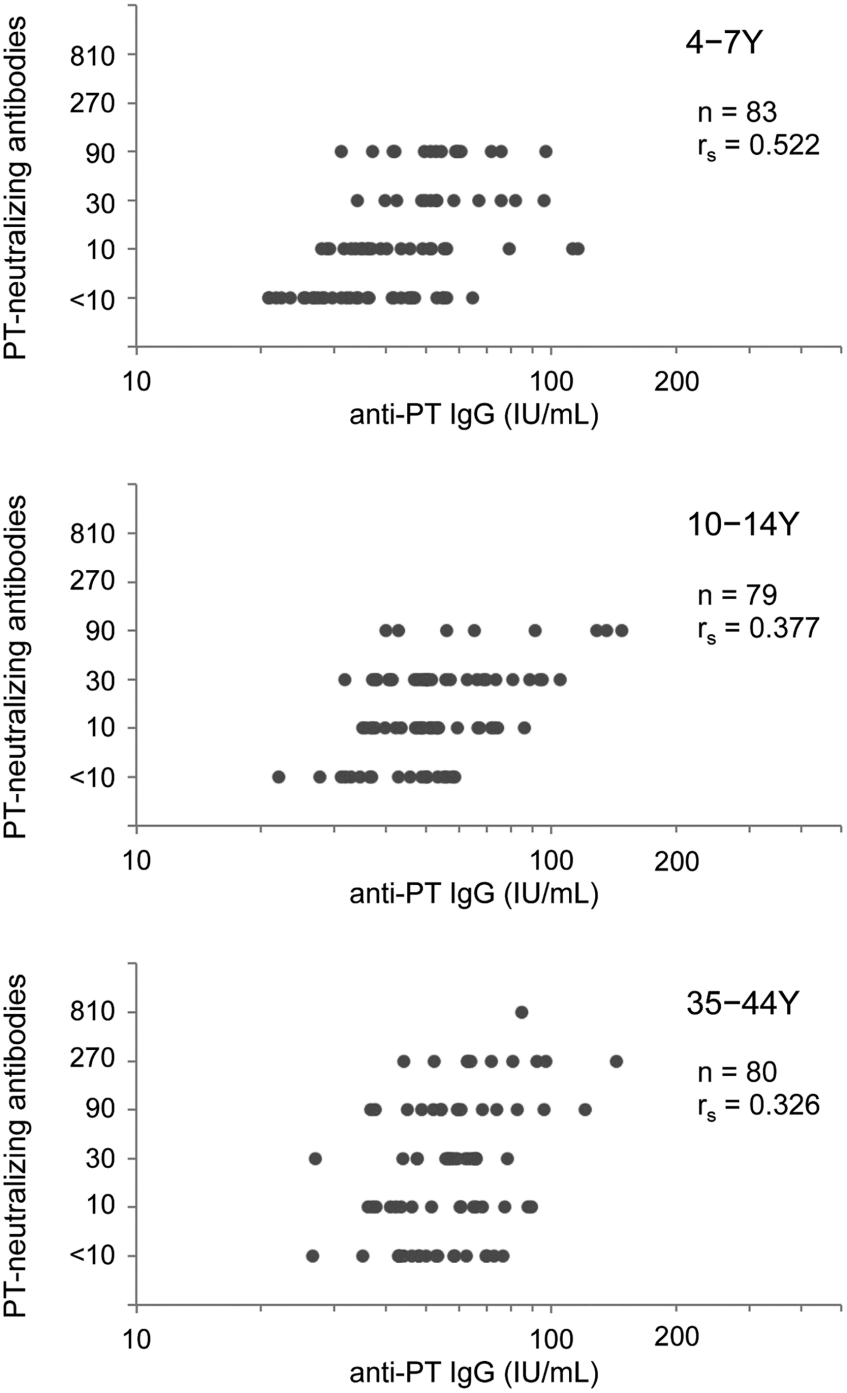
Correlations between anti-PT IgG and PT-neutralizing antibody titers among young children, older children, and adults. Anti-PT IgG titers in individual serum samples (x-axis in log scale) were plotted against PT-neutralizing antibody titers (y-axis). Values on the y-axis indicate the reciprocal serum dilution required to neutralize PT activity. The age groups were 4–7 years, 10–14 years, and 35–44 years. r_s_, Spearman’s rank correlation coefficient; Y, years.

A total of 242 serum samples were tested for both anti-PT IgG and PT-neutralizing antibodies: 83 samples from young children, 79 samples from older children, and 80 samples from adults. As shown in Fig 2, all age groups exhibited positive correlations between the titers of anti-PT IgG and PT-neutralizing antibodies, although this correlation decreased with age. The Spearman’s rank correlation coefficients (r_s_) were 0.522 (*p* < 0.01), 0.377 (*p* < 0.01), and 0.326 (*p* < 0.01) for young children, older children, and adults, respectively. This demonstrates that the non-IgG immunoglobulins in the PT-neutralizing antibodies may increase with age.

### Ratios of PT-neutralizing antibodies to anti-PT IgG

**Fig 3:**
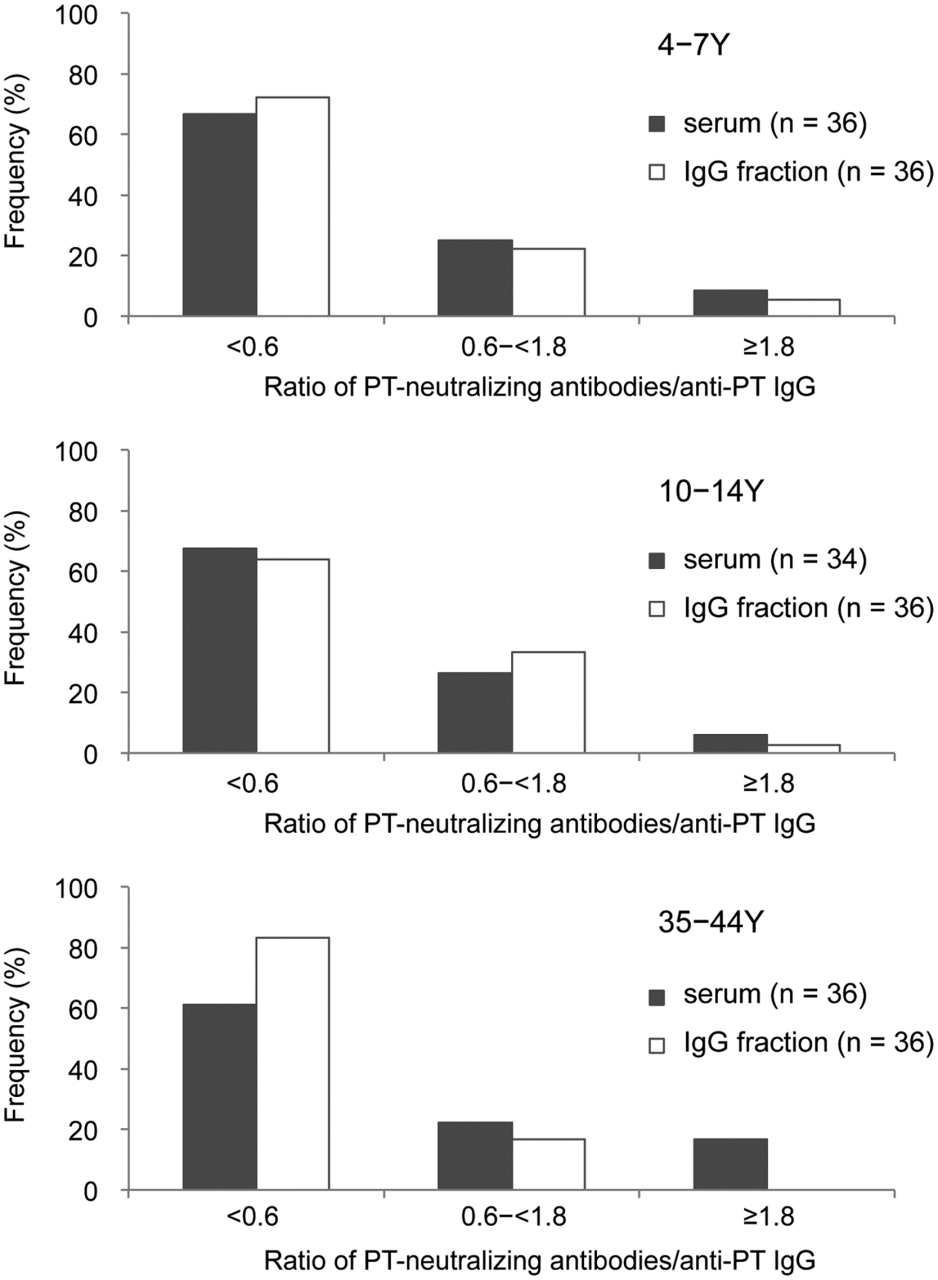
Comparing the ratio of PT-neutralizing antibodies to anti-PT IgG between the serum and purified IgG fractions from young children, older children, and adults. The IgG fractions were purified from serum samples that were collected in 2014, and the ratios of PT-neutralizing antibodies (titer) to anti-PT IgG (IU/mL) were compared. The age groups were 4–7 years, 10–14 years, and 35–44 years. Numbers in parentheses indicate the number of samples that were analyzed. Y, years. The frequencies of the serum and IgG fraction ratios were significantly different among the adults (*p* = 0.016), but not among young and older children (*p* > 0.05), indicating that the adults with the highest ratio ≥1.8 had non-IgG anti-PT immunoglobulins.

To assess the involvement of non-IgG immunoglobulins in the PT-neutralizing antibodies, we purified IgG from serum samples that were collected in 2014, and measured the titers of both anti-PT IgG and PT-neutralizing antibodies in the IgG fraction. The IgG fractions were classified according to the ratio of PT-neutralizing antibodies (titer) to anti-PT IgG (IU/mL), categorized as <0.6, 0.6–<1.8, and ≥1.8. Several serum samples obtained from adults had a high ratio (≥1.8) (S3 Fig). The distribution of these PT-neutralizing antibodies-to-anti-PT IgG ratios did not differ significantly when measured in the serum and in the purified IgG samples obtained from young and older children (*p* > 0.05) (Fig 3). In contrast, significantly different distributions were observed between the adult serum and purified IgG samples (*p* = 0.016), probably due to the low frequency of the high ratio (≥1.8) category in the purified IgG samples. The IgG fractions of adult serum samples exhibited a low frequency (0%) of the highest ratio (≥1.8) and a high frequency (83.8%) of the lowest ratio (<0.6).

We also analyzed the antibody titer ratios (PT-neutralizing antibodies/anti-PT IgG) from 242 serum samples that were collected during 2013–2014: 83 samples from young children, 79 samples from older children, and 80 samples from adults (S4 Fig). The frequencies of the ratios were not significantly different when we compared the serum samples from 2013–2014 and 2014 (*p* > 0.576), which suggested that there was no apparent bias related to our use of the serum samples that were collected in 2014.

### Avidities of the anti-PT IgG and anti-FHA IgG

**Fig 4:**
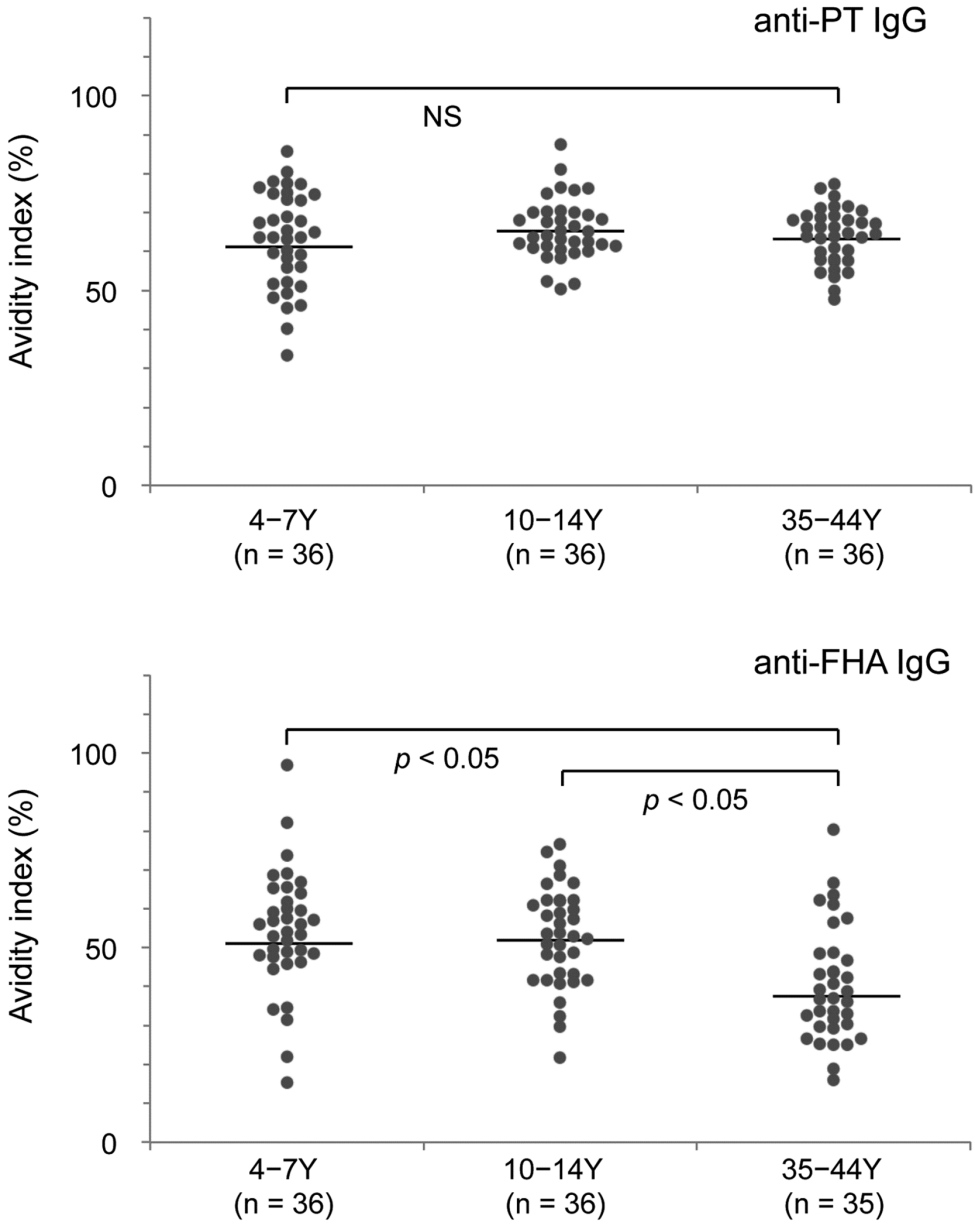
Avidities of anti-PT IgG and anti-FHA IgG among young children, older children, and adults. The avidity indexes (AI) were determined in IgG fractions that were purified from serum samples, which were collected in 2014. The age groups were 4–7 years, 10–14 years, and 35–44 years. Bars indicate the geometric mean AI values. Numbers in parentheses indicate the number of samples that were analyzed. Y, years; NS, not significant. *P*-values were determined using the Steel-Dwass test.

To assess the quality of the pertussis antibodies, we evaluated the avidities of the anti-PT IgG and anti-FHA IgG using the IgG fractions. As shown in Fig 4, all age groups (young children, older children, and adults) exhibited high geometric mean values for the anti-PT IgG AIs (61.8–65.3%), and there was no significant age-related difference in the AI values (*p* > 0.05). In contrast, the age-specific AI values for anti-FHA IgG were significantly different (*p* < 0.05), with adults exhibiting lower AI values: 51.7% for young children, 51.0% for older children, and 37.9% for adults.

In all age groups, the AI value for anti-PT IgG was not correlated with the anti-PT IgG titers (r_s_ = –0.110 to –0.136, *p* > 0.05). Similarly, the AI value for anti-FHA IgG was not correlated with the anti-FHA IgG titers among young and older children (r_s_ = 0.183 and 0.138, *p* > 0.05). However, a weak positive correlation was observed among adults (r_s_ = 0.388, *p* < 0.05).

## Discussion

In the present study, we confirm that 35–44-year-old adults had high seroprevalences of both anti-PT IgG and PT-neutralizing antibodies compared to young and older children, although an adolescent booster dose of pertussis vaccine is not included in the national immunization schedule of Japan. Our CHO cell clustering assay also demonstrated that some adults had both anti-PT IgG and non-IgG anti-PT immunoglobulins. Moreover, our avidity ELISA assay revealed that the adults had high-avidity anti-PT IgG, which were also observed among the young and older children. Although some adults had non-IgG anti-PT immunoglobulins, the quality of the anti-PT IgG in adult samples was the same as that determined by the avidity ELISA in the samples obtained from children. The children and adults did not exhibit significantly different seroprevalences of anti-FHA IgG, although the adults had significantly lower avidity anti-FHA IgG (AI: 37.9%), which suggests that there are age-related differences in the quality of anti-FHA IgG. To the best of our knowledge, this is the first study to compare the quantity and quality of pertussis antibodies among children and adults.

To determine the cause of the high seroprevalence of anti-PT IgG among adults, we analyzed the correlation between anti-PT IgG and PT-neutralizing antibodies among adults, and compared the results to those from among young and older children. All age groups exhibited positive correlations between the antibody titers, although the correlation decreased with age (Fig 2). A previous study also revealed a good correlation among vaccinated individuals [16], although another study revealed that the correlation was not strong for patients with pertussis [17]. In the present study, adults exhibited a lower correlation (vs. that among young and older children), which indicates that the high seroprevalence among adults might be related to natural infection with *B*. *pertussis*. This concept is supported by our observation that some adults had non-IgG anti-PT immunoglobulins (e.g., anti-PT IgM and IgA) (Fig 3). In this context, it is known that natural infection with *B*. *pertussis* not only induces production of serum IgG against *B*. *pertussis* proteins, but also induces production of IgA and IgM antibodies [25]. A large pertussis epidemic occurred in Japan during the late 2000s, and the incidence among adolescents and adults reached 52.9% of all reported cases in 2010 [5]. Recently, it was reported that anti-PT IgG titer changed very little over a long period time (≥1 year) among Japanese adults [26]. Therefore, the high seroprevalence of pertussis antibodies among adults might be related to the pertussis epidemic. Furthermore, a high seroprevalence of anti-PT IgG has been observed among adults in several countries since 2000 [8, 10, 11, 13, 27]. These findings support our interpretation that a pertussis epidemic can cause an increase in the seroprevalence of anti-PT IgG among adults.

In the present study, we found that the seroprevalences of anti-FHA IgG were not significantly different when we compared young children, older children, and adults. This result agrees with serosurveillance data from the 2013 NESVPD (http://www.nih.go.jp/niid/ja/y-graphs/1600-yosoku-index-e.html), and previous studies have also demonstrated that the seroprevalence of anti-FHA IgG tends to remain constant or increase with age [13, 15]. A possible explanation for this pattern is that the antibody response to FHA is not specific to *B*. *pertussis* infection. Unlike anti-PT IgG which would be produced specifically after contact with *B*. *pertussis*, anti-FHA IgG appear not only after contact with *B*. *pertussis* but could also be induced by contact with the human respiratory pathogens, *Bordetella parapertussis*, *Haemophilus influenzae*, and *Mycoplasma pneumoniae* [25, 28–31]. Exposure to these human pathogens can produce anti-FHA-like protein(s), which may induce cross-reactive antibodies against *B*. *pertussis* FHA (i.e., low-avidity antibodies to FHA). Therefore, we speculate that adults with low-avidity anti-FHA IgG had been previously infected (symptomatically or asymptomatically) with *B*. *parapertussis*, *H*. *influenzae*, or *M*. *pneumoniae*, although the natural infection rates of the pathogen(s) during adulthood are unknown. Our study provides the first demonstration of low-avidity anti-FHA IgG among adults.

Avidity represents a functional measure of antibody affinity maturation. A previous study has demonstrated that the AI of anti-PT IgG among adolescents and adults was higher after natural infection with *B*. *pertussis*, compared to after booster vaccination with an ACV, and that healthy individuals had low-avidity anti-PT IgG [20]. Interestingly, the present study did not reveal any significant differences in the AIs of anti-PT IgG among young children, older children, and adults (AI: 61.8–65.3%), although older children have a high risk of *B*. *pertussis* infection in Japan [32]. Thus, it remains unclear why young and older children exhibited similar AIs for anti-PT IgG. Our AI assay was performed using purified IgG, and would not have been affected by non-IgG anti-PT immunoglobulins, which supports the reliability of our AI results. Nevertheless, there are few reports regarding the quality of anti-PT IgG and anti-FHA IgG among healthy individuals, and further studies are needed to assess the quality of pertussis antibodies among healthy individuals.

In conclusion, 35–44-year-old Japanese adults exhibited a high seroprevalence of anti-PT IgG during 2013–2014, and both adults and children had high-avidity anti-PT IgG. However, the adults had lower-avidity anti-FHA IgG, compared to the children. Therefore, our findings suggest that the adults had been infected with *B*. *pertussis* and other pathogens (e.g., *B*. *parapertussis*, *H*. *influenzae*, or *M*. *pneumoniae*) during their adulthood, although further studies are needed to test this hypothesis.

## Supporting information

S1 Fig Reproducibility of CHO cell clustering assay for PT-neutralizing antibodies. Three independent experiments were performed on 4 reference sera: JNIH-10, Japanese reference serum (anti-PT IgG, 300 IU/mL); negative control S4, serum of a healthy infant aged 7 months (153 IU/mL); negative control S5, serum of a healthy infant aged 8 months (255 IU/mL); and positive control M14, serum of a patient with pertussis, aged 4 years (2,800 IU/mL).
(TIF)

S2 Fig Reproducibility of avidity ELISA for anti-PT IgG and anti-FHA IgG. Three independent experiments were performed on 4 reference sera: JNIH-10, Japanese reference serum (300 IU/mL for anti-PT IgG; 400 IU/mL for anti-FHA IgG); negative control S4, serum of a healthy infant aged 7 months (153 IU/mL; 188 IU/mL); negative control S5, serum of a healthy infant aged 8 months (255 IU/mL; 100 IU/mL); positive control M14, serum of a patient with pertussis, aged 4 years (2,800 IU/mL; 300 IU/mL). The positive control M14 was assayed with a 1:2000 dilution for anti-PT IgG, and with a 1:400 dilution for anti-FHA IgG. Other sera were assayed with 1:200 dilutions for both anti-PT IgG and anti-FHA IgG. The maximum coefficients of variation were 8.1% and 13.3% for anti-PT IgG and anti-FHA IgG, respectively.
(TIF)

S3 Fig Ratio of PT-neutralizing antibodies to anti-PT IgG in the serum samples obtained from young children, older children, and adults. A total 242 serum samples collected during 2013–2014 were analyzed: 83 samples from young children (4–7 years old), 79 samples from older children (10–14 years old), and 80 samples from adults (35–44 years old). The ratios of PT-neutralizing antibody (titer) to anti-PT IgG (IU/mL) are plotted. The serum samples with PT-neutralizing antibody titer of <10 were calculated as the ratio of <0.6.
(TIF)

S4 Fig Comparing the ratio of PT-neutralizing antibodies to anti-PT IgG among young children, older children, and adults. A total of 242 serum samples were collected during 2013–2014 and were analyzed: 83 samples from young children (4–7 years old), 79 samples from older children (10–14 years old), and 80 samples from adults (35–44 years old). The distributions in adult serum samples were significantly different to those from young and older children (each, *p* < 0.05, Fisher’s exact test). Y, years.
(TIF)
