# Supplementary figures and images for: A high seroprevalence of antibodies to pertussis toxin among Japanese adults: Qualitative and quantitative analyses
Source: PLoS One. 2017 Jul 10;12(7):e0181181. doi: 10.1371/journal.pone.0181181 (PMC5507317; doi:10.1371/journal.pone.0181181)

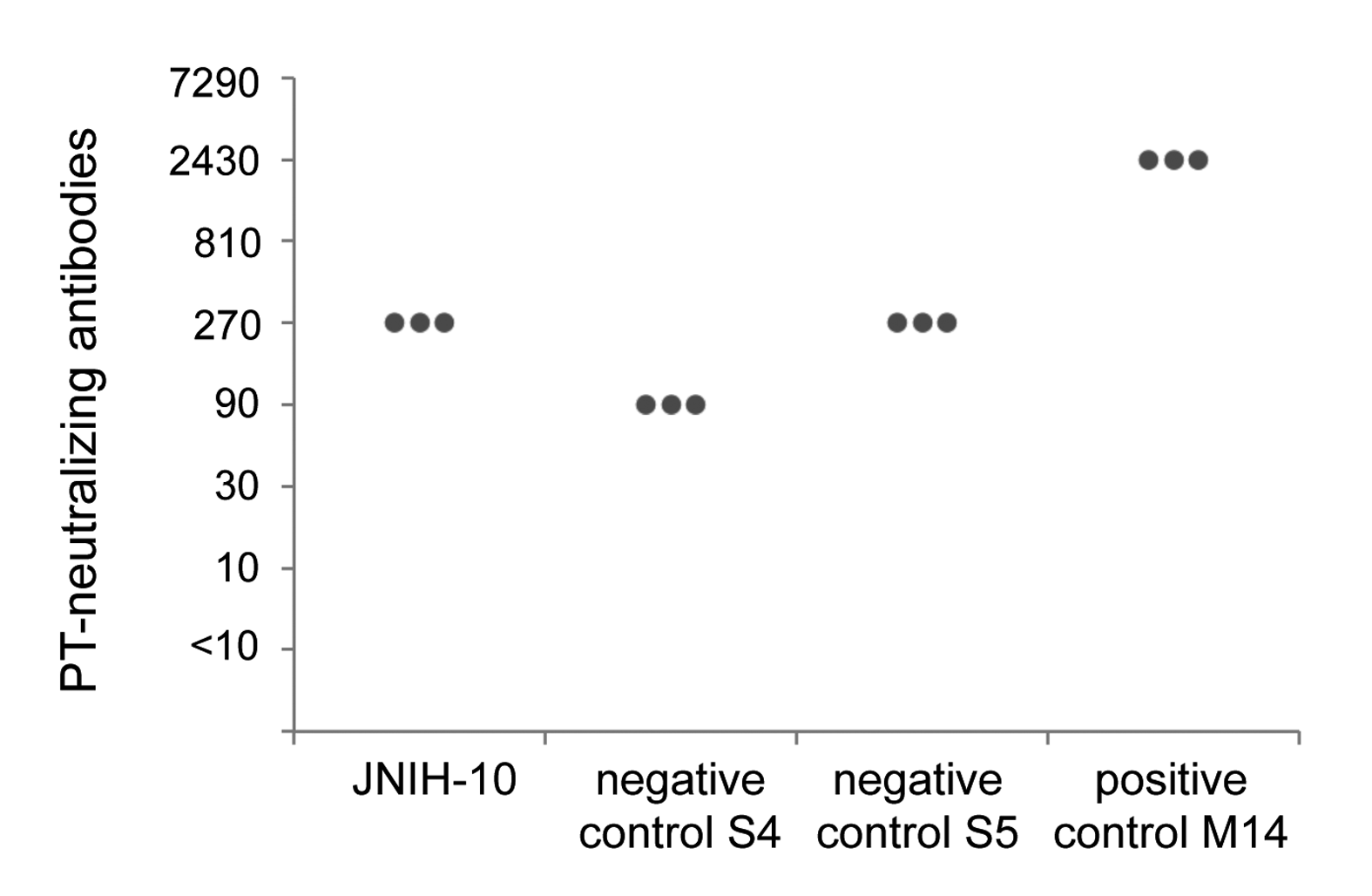

Supplement: S1 Fig — Three independent experiments were performed on 4 reference sera: JNIH-10, Japanese reference serum (anti-PT IgG, 300 IU/mL); negative control S4, serum of a healthy infant aged 7 months (153 IU/mL); negative control S5, serum of a healthy infant aged 8 months (255 IU/mL); and positive control M14, serum of a patient with pertussis, aged 4 years (2,800 IU/mL). (TIF) [file pone.0181181.s001.tif]

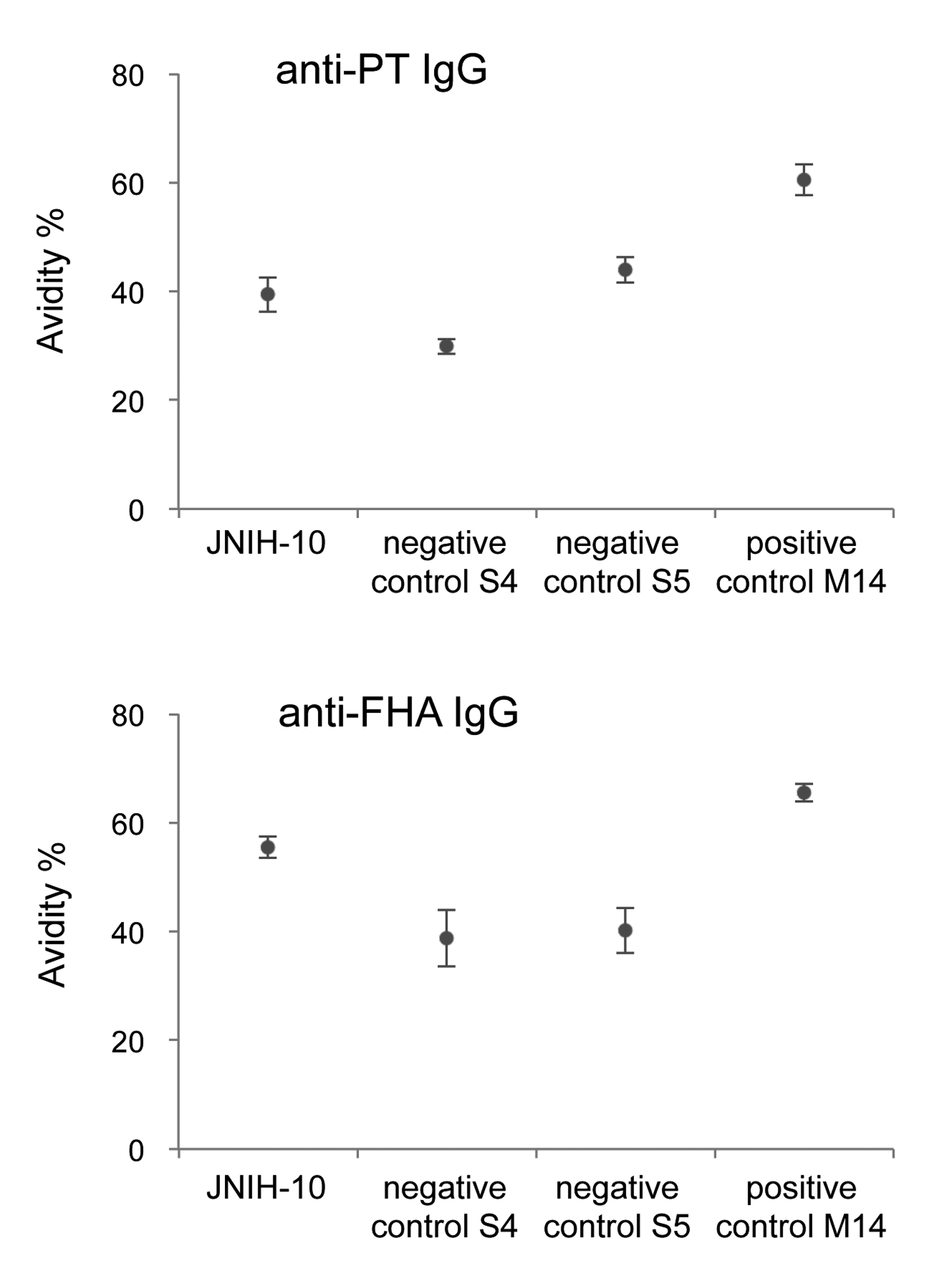

Supplement: S2 Fig — Three independent experiments were performed on 4 reference sera: JNIH-10, Japanese reference serum (300 IU/mL for anti-PT IgG; 400 IU/mL for anti-FHA IgG); negative control S4, serum of a healthy infant aged 7 months (153 IU/mL; 188 IU/mL); negative control S5, serum of a healthy infant aged 8 months (255 IU/mL; 100 IU/mL); positive control M14, serum of a patient with pertussis, aged 4 years (2,800 IU/mL; 300 IU/mL). The positive control M14 was assayed with a 1:2000 dilution for anti-PT IgG, and with a 1:400 dilution for anti-FHA IgG. Other sera were assayed with 1:200 dilutions for both anti-PT IgG and anti-FHA IgG. The maximum coefficients of variation were 8.1% and 13.3% for anti-PT IgG and anti-FHA IgG, respectively. (TIF) [file pone.0181181.s002.tif]

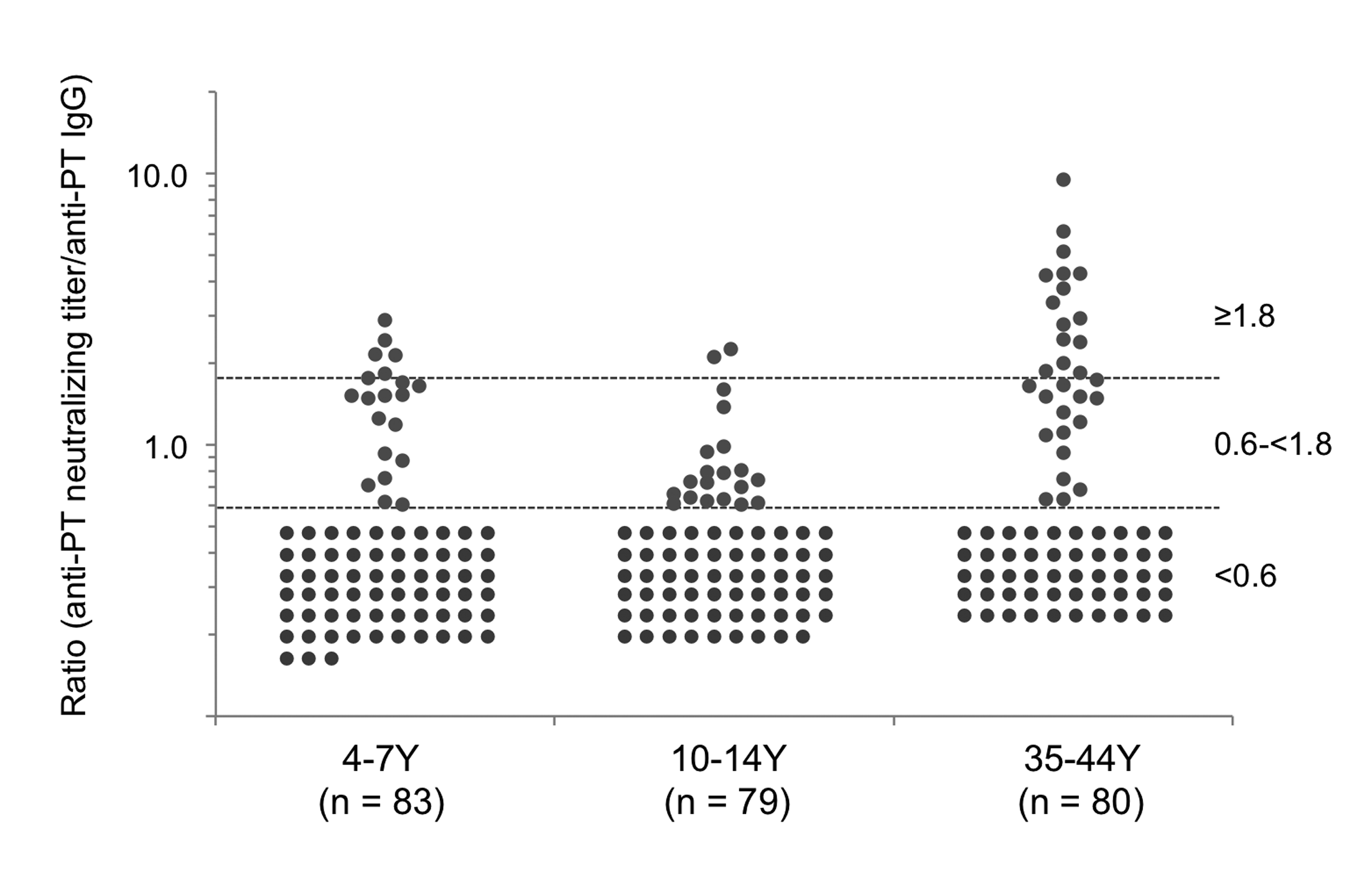

Supplement: S3 Fig — A total 242 serum samples collected during 2013–2014 were analyzed: 83 samples from young children (4–7 years old), 79 samples from older children (10–14 years old), and 80 samples from adults (35–44 years old). The ratios of PT-neutralizing antibody (titer) to anti-PT IgG (IU/mL) are plotted. The serum samples with PT-neutralizing antibody titer of <10 were calculated as the ratio of <0.6. (TIF) [file pone.0181181.s003.tif]

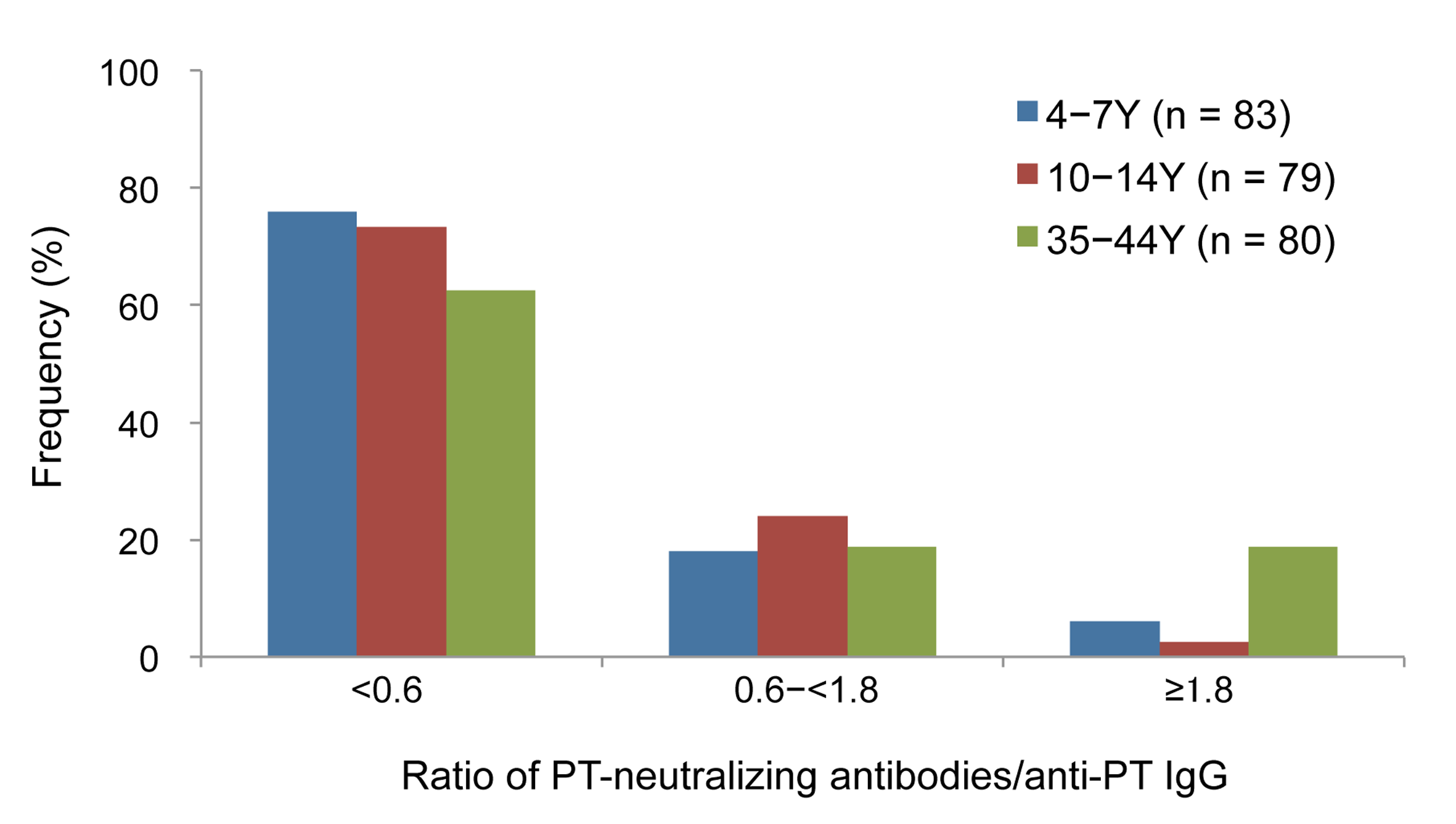

Supplement: S4 Fig — A total of 242 serum samples were collected during 2013–2014 and were analyzed: 83 samples from young children (4–7 years old), 79 samples from older children (10–14 years old), and 80 samples from adults (35–44 years old). The distributions in adult serum samples were significantly different to those from young and older children (each, p < 0.05, Fisher’s exact test). Y, years. (TIF) [file pone.0181181.s004.tif]
